# Quartz Crystal Microbalance With Dissipation Monitoring: A Powerful Method to Predict the *in vivo* Behavior of Bioengineered Surfaces

**DOI:** 10.3389/fbioe.2018.00158

**Published:** 2018-10-30

**Authors:** Chiara Tonda-Turo, Irene Carmagnola, Gianluca Ciardelli

**Affiliations:** ^1^Department of Mechanical and Aerospace Engineering, Politecnico di Torino, Turin, Italy; ^2^POLITO BIOMedLAB, Politecnico di Torino, Turin, Italy; ^3^Department for Materials and Devices of the National Research Council, Institute for the Chemical and Physical Processes (CNR-IPCF UOS), Pisa, Italy

**Keywords:** quartz crystal microbalance with dissipation monitoring (QCM-D), cell adhesion, cell death, cell cytoskeleton, bioengineered surface characterization

## Abstract

The Quartz Crystal Microbalance with dissipation monitoring (QCM-D) is a tool to measure mass and viscosity in processes occurring at or near surfaces, or within thin films. QCM-D is able to detect extremely small chemical, mechanical, and electrical changes taking place on the sensor surface and to convert them into electrical signals which can be investigated to study dynamic process. Surface nanotopography and chemical composition are of pivotal importance in biomedical applications since interactions of medical devices with the physiological environment are mediated by surface features. This review is intended to provide readers with an up-to-date summary of QCM-D applications in the study of cell behavior and to discuss the future trends for the use of QCM-D as a high-throughput method to study cell/surface interactions overcoming the current challenges in the design of biomedical devices.

## Introduction

Quartz crystal microbalance with dissipation monitoring (QCM-D) is an easy, highly versatile as well as highly sensitive method to study processes occurring at the surfaces or within thin films. QCM was introduced in 1960s to monitor layer formation in vacuum and air thanks to the high mass sensitivity of the 5 MHz quartz system (0.057 Hz cm^2^ ng^−1^) which was superior compared with other available technologies (Janshoff et al., 2000). Then, it gained interest in biomedical application since 1980s when novel QCM systems able to work in liquid media was introduced (Marx, 2003). However, the QCM has achieved resounding success since 1995 when a new system able to measure changes in damping properties of adsorbed layers (QCM-D) were developed, patented and commercialized by Q-Sense (Rodahl et al., 1995; Rodahl, 1996).

**Figure 1:**
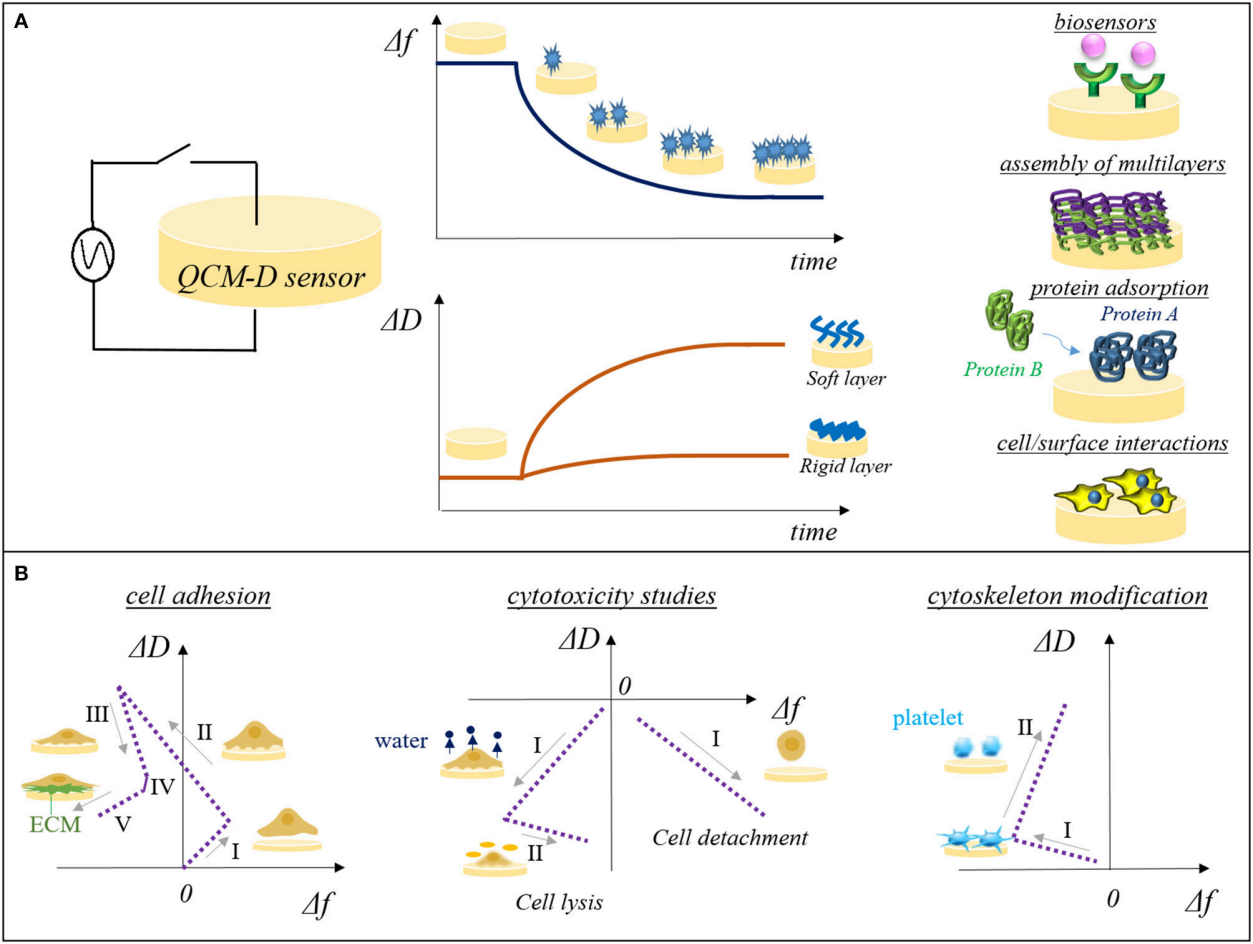
**(A)** Schematic representation of QCM-D plots reporting frequency factor (Δ*f*) and dissipation energy factor (Δ*D*) vs. time. QCM-D system is mainly applied in the study of binding efficacy of probing surface of biosensors, assembly of multilayers, competitive protein adsorption studies, and recently for cell/surface interactions. **(B)** Representative Δ*D-*Δ*f* plot signatures for cell adhesion on sensor surfaces (I-initial adhesion, II-formation of attachment points, III-cell spreading, IV-steady state of spread cells, V-production of ECM), cytotoxicity studies (left side: I-water release from cytoskeleton, II-cell lysis; right side: I-cell detachment) and cytoskeleton modification such as platelets activation (I-initial interactions among surface sensor and platelets, II-platelets spreading and pseudopodia formation).

To date, QCM-D can be applied to monitor processes in real-time by measuring changes in frequency and energy dissipation of the system composed by the surface or thin film of interest adsorbed on a piezoelectric quartz sensor. Quartz sensors are applied in the QCM-D devices as a mechanical oscillation of characteristic frequency can be produced on the crystal by applying an alternated electric field. When a change on the mass occurred at the surface interface or within the thin film, a frequency shift from the fundamental resonant frequency of the crystal (Δ*f*) is measured making the QCM-D a quantitatively and ultrasensitive technology to measure mass changes. In addition to Δ*f*, QCM-D simultaneously monitors the viscoelastic properties of the overlayer or thin film adsorbed to the quartz sensors by recording changes in the energy dissipation factor (*D*) (Figure 1A). When the generator is switched off the crystal oscillation amplitude decays exponentially and the energy dissipation factor can be measured by recording the amplitude of the oscillation as a function of time (Rodahl et al., 1995). Higher are the dissipative properties of the overlayer faster is the decay time resulting in substantial energy dissipation factor shift (Δ*D*) from rigid state.

In the case of rigid layer (Δ*D* = 0), when the change in mass occurs in air or in vacuum, the frequency shift (Δ*f*) is proportional to mass change and it can be quantified applying the Sauerbrey equation (Sauerbrey, 1959). However, biological processes take place in liquid environment where the Sauerbery equation is no more valid and the equation described by Kanazawa and Gordon has to be used to relate Δ*f* to mass changes (Kanazawa and Gordon, 1985). For not rigid layers where the damping of the layer is significant (Δ*D* > *0*), the Voight model is applied to analyze collected data and equations developed by Voinova et al. (1998) provide information on the mass and viscoelastic properties of the viscous layer. Latest QCM-D devices allow for simultaneously recording of Δ*f* and Δ*D* for multiple harmonics (up to the 13th overtone) which permit to better extract the adsorbed overlayer characteristics (such as density, storage, and loss moduli; Hovgaard et al., 2007). An historical perspective describing the QCM-D working principles and its applications to study complex systems was recently published by Chen et al. (2016).

In the past 15 years, QCM-D has been increasingly used as a tool to characterize the biological interactions of engineered surfaces. To date, QCM-D crystals have been mainly applied to monitor the binding efficacy of probing surface of biosensors (Chen et al., 2010; Xu et al., 2011; Ertekin et al., 2016) as well as to verify the biofunctionalization of nanoengineered surfaces through self-assembled monolayers (SAMs; Minsky et al., 2016) or layer-by-layer (LbL) technologies (Chen et al., 2010). In addition, proteins adsorption competitive studies on biomedical device surfaces have been performed thanks to the possibility to measure at the same time the mass and the viscoelastic properties of absorbed biomolecules, which can be related to protein conformation. Protein adsorption kinetics on polyHEMA (Teichroeb et al., 2008), stainless steel (Chandrasekaran et al., 2013), and titanium oxide (Pegueroles et al., 2012) were analyzed to study interaction between biomedical devices and biological fluids after implantation. Furthermore, QCM-D has been used to assess the antifouling properties of surfaces such those composed of branched polymers (Luan et al., 2017). Besides composition and nanoscale chemistry, different surface morphologies were investigated to relate nanostructures to protein adsorption kinetics (Hovgaard et al., 2008; Chandrasekaran et al., 2013).

Phenomena occurring at the interface within surfaces and biological fluids are fundamental to address specific cell response on biomedical surfaces. QCM-D allows to study these phenomena in real-time without requiring labeling processes which could interfere with the system, thus preventing any loss of information (Nowacki et al., 2014). Furthermore, QCM-D experimental setup is equipped with a temperature-controlled system able to mimic the physiological conditions within the equipment chamber. In the last 5 years a growing interest on QCM-D related to cell behavior studies has been observed as this technique appears to be particularly well-suited for real-time acquisition of cell responses and to monitor cytoskeleton changes after internal or external stimuli. Recently, novel crystal chamber set-ups have been commercialized to permit a multiple characterization of the processes occurring on the crystal surface. Among others, the open window chamber, which combined traditional QCM-D measurements with light microscopy, and the ellipsometric modulus are applied to monitor cell-surface interactions as they allow to follow in real-time changes in cell morphology (Marcus et al., 2012; Tymchenko et al., 2012) and in overlayer thickness (van der Meulen et al., 2014), respectively.

The purpose of this article is to provide a summary of the present state of the art of QCM-D as a tool to monitor cell behavior on surfaces. Then, the future trends in the use of QCM-D as a tool to study phenomena occurring at surface/cell interface, which are pivotal for innovation in the biomedical field, are envisioned.

## QCM-D as a tool to monitor cell adhesion

**Figure 2:**
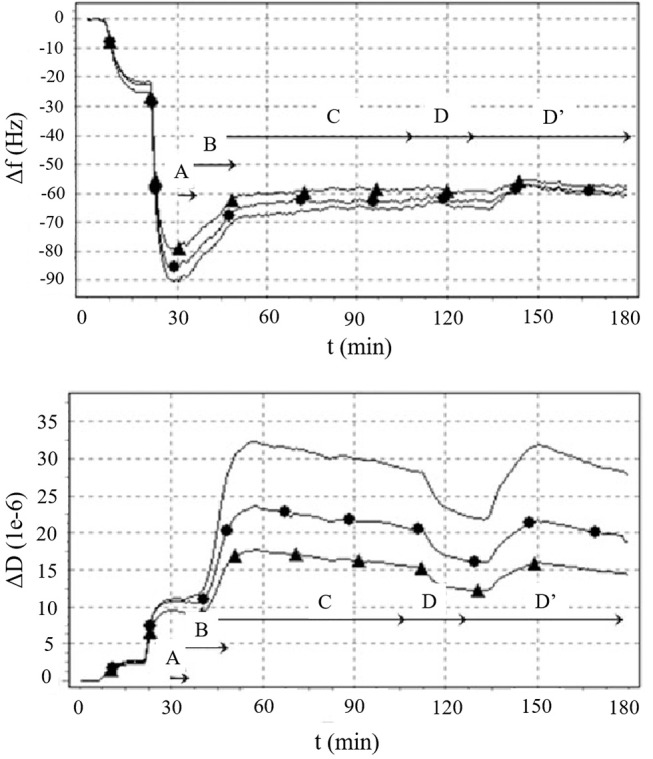
The Δ*f* and Δ*D* signals (3rd-line, 5th-circle, and 7th-triangle overtones) over time of NIH3T3 fibroblasts cultured on a collagen I functionalized sensor. Five events were identified: (A) cell seeding, (B) cell adhesion, (C) cell spreading, (D) cytoskeleton rearrangement after addition of the cytoskeleton-perturbing agent cytochalasin D (2 μg/ml) and (D′) cell recovery after cytochalasin D removal. Reproduced with permission from Tymchenko et al. (2012). The different phases of cell/surface interaction were confirmed by real-time monitoring of cell morphology and distribution through polarized light microscopy.

QCM-D sensors was applied in the study of cell-surface interactions as the piezoelectric mass sensing combined with the monitoring of dissipation changes was related to different colonization stages of cells on surfaces (as attachment, adhesion, and spreading; Saitakis and Gizeli, 2012; Tymchenko et al., 2012). When a cell is interacting with the sensor surface a shift in the Δ*f* can be detected, however the majority of the information on cell state is provided by the energy dissipation factor (Δ*D*), index of the fact that the signal is mainly attributable to the viscoelastic behavior of the cell part adhering on the crystal. This is mainly ascribed to the size of cells, which overcome the sensitive limit of the instrument. Indeed, the QCM-D penetration signal is ~0.25 μm in water, allowing the detection of only a fraction of the adhered cell size and consequently effects on frequency shift are mainly associated to adhesion contact points located on the cell membranes (Saitakis and Gizeli, 2012). The contact of the cells with the substrate induces a small decrease of Δ*f* which is not proportional to the real mass adsorbed while in contrast Δ*D* strongly increases having higher shift when adhesion involved a small fraction of the cell membranes (low spreading and limited numbers of focal contact points). During the cell spreading no significant changes are detected in frequency shift compared to adhesion, whereas dissipation slightly decrease due to the rearrangement of cytoskeleton and a subsequent cell stiffening (Tymchenko et al., 2012) responsible for the decrease in damping events (Figure 2). The information collected from the temporal plot of Δ*f* and Δ*D* can be combined in a Δ*D-*Δ*f* plot. In this contest, Δ*D-*Δ*f* plot (frequency shift plotted vs. energy dissipation shift) provides greater insight into changes in the structural conformation of cells and it is used to monitor the different stages of the cell adhesion: from initial cell/surface interactions (initial adhesion, formation of attachment points) to the following changes in the cytoskeleton conformation characteristics of cell spreading. Westas et al. (2015) monitored the interaction of human gingival fibroblasts on osteoinductive surfaces functionalized with hydroxyapatite nanoparticles when a complete coverage of the sensor surface was achieved. Five consecutive stages were identified by analyzing the recorded QCM-D signals: (i) initial adhesion when cells were close to the surface and started making few attachment points [Δ*f* increased as cells behaving like a coupled oscillator (Granéli et al., 2004) and Δ*D* slightly increased], (ii) the number of adhered cells increased as well as the attachment points (Δ*f* decreased as the number of attachment point increased and a more homogenous layer is formed at the sensor interface causing Δ*D* increase), (iii) cell spreading occurred (Δ*D* rapidly decreased due to the formation of mature focal adhesion complex and Δ*f* decrease as the number of mature focal adhesion complex was lower than the initial adhesion points formed at the second stage), (iv) steady state of spread cells (Δ*f* and Δ*D* decreased slowly), and (v) production of extracellular matrix (ECM) from adhered cells (Δ*f* decreased due to new mass on the crystal; Figure 1B).

The real-time monitoring of cells behavior on surfaces was recently exploited in the optimization of implantable surfaces in order to design *ad-hoc* substrates having chemical-physical features able to enhance cell adhesion. As the electric charge of the surface material is crucial for the integration in the biological environment, Kao et al. (2017) employed the potential of QCM-D equipment to follow the NIH3T3 mouse embryonic fibroblast adhesion on surface with different ζ-potentials. Higher ζ-potential surfaces allowed the direct interaction between cells and surface resulting in a high adhesion and cell spreading while the electrostatic repulsions among surface and cells occurring in presence of lower ζ-potentials required the formation of an ECM-like layer prior to cell adhesion and spreading. Kushiro et al. (2016) used the same approach to monitor the cell adhesion process on various SAM surfaces. Interestingly, QCM-D signals allowed to distinguish from different material-cell interactions that were not detected by morphological analysis using confocal microscopy. These findings confirmed the strength of QCM-D setup in the detection of cell changes at the micro and nanoscale, which cannot be measured through conventional methods. Furthermore, Chronaki et al. (2016) employed QCM-D to analyze the difference between the adhesion pattern of normal thyroid and anaplastic thyroid cancer cells demonstrating that starting from dissipation over frequency curves it is possible to accurately discriminate cancer cell from healthy ones, envisioning the use of QCM-D as a promising diagnostic tool.

In addition to cell, bacteria-surface interactions were monitored using QCM-D device (Reipa et al., 2006; Olsson et al., 2010, 2011). Bacteria adhesion is a highly studied topic as adhesion of bacteria on biomedical device surfaces results in biofilm formation, which is one of the more frequent cause of implant failure. QCM-D was applied in the attempt to monitor the antibacterial efficacy of two well-known aminoglycosides, kanamycin A, and neomycin B (Joshi et al., 2015). The modeling of frequency and dissipation energy shifts allowed to get detailed knowledge on mechanism of action of different aminoglycosides in bacteria membrane disruption.

## QCM-D as a tool to monitor cytotoxicity and cell viability

Cells are highly sensitive to perturbations in the surrounding environment and changes interfering with the cell activities can lead to cell death. The monitoring of cell behavior when cells are in contact with bioengineered surfaces, biomaterials, or drugs is fundamental to identify cytotoxic or non-cytotoxic effects. Traditional methodologies to study cytotoxicity and cell viability are based on microscopy and flow cytometry which are unable to monitor cell health *in situ* and in real-time. Hence, the ability of QCM-D signals to detect cytoskeletal changes can be exploited to assess the cell health in response to external perturbations. Fatisson et al. (2011a) investigated the cellular response during exposure to two different cytomorphic agents (Triton-X 100 and lipopolysaccharide—LPS) on bovine aortic endothelial cells (BAECs) adhering on QCM-D sensor. QCM-D sensors were pre-incubated with BAECs, then coated sensors were mounted into the device chamber and the chemicals were injected in the chamber flowing on the cells attached to the sensor. Both changes in frequency and in energy dissipation were recorded to model cytoskeleton changes in real time. Δ*D-*Δ*f* plots revealed two different cell death mechanisms induced by the two cytomorphic agents. High concentrations of Triton-X 100 led to cell lysis, as confirmed by the Δ*D-*Δ*f* plot. In this plot, a first phase of Δ*f* and Δ*D* drop was detected suggesting mass adsorption and more rigid layer formation (probably ascribed to Triton-X 100 micelles adsorption and water release from cytoskeleton), followed by the effective cell lysis which induced a Δ*f* increase and a slow Δ*D* drop (Figure 1B). On the other hand, LPS exposure resulted in a single slope of the Δ*D-*Δ*f* plot with a steady increase of Δ*f* and decrease of Δ*D*. LPS induced a progressive detachment of the BEACs from the QCM-D sensor (from spread cells to round cells) which resulted in mass loss and reduction of damping. Kandel et al. (2014) assessed the viability of human umbilical vein endothelial cells (HUVECs) on different fibronectin-coated QCM-D sensors monitoring the cytoskeleton changes in presence or absence of an actin depolymerizing agent.

Further evidences of the ability of QCM-D to sense cell death were described by Nowacki et al. (2014) which investigated QCM-D signal changes when human breast cancer cells (MCF-7) adhering on quartz crystal surfaces were treated with staurosporine (STS), a protein kinase inhibitor known to induce cell apoptosis. Δ*D-*Δ*f* plot displayed a simultaneous increase of Δ*f* and a decrease of Δ*D*, which is typical of apoptosis-mediated cell death.

These findings pave the way for the use of QCM-D not only in the study of cell cytotoxicity but also as a tool to investigate the efficacy of drugs in fighting cancer or other diseases where selective cell death is targeted. The high sensitivity of QCM-D and its ability to continuously monitor the events occurring on the quartz sensors allow to distinguish between the various cell death subtypes and pathways which are fundamental aspects for drug efficacy evaluation (Kepp et al., 2011). Pioneering work of Zhang et al. tracked changes in frequency and energy dissipation of MCF-7 cells treated with resveratrol reporting the use of QCM-D as a cancer drug screening technology for the first time (Zhang et al., 2015a).

## QCM-D as a tool to monitor important phenomena in cells

The opportunity to detect in real time the rearrangement of cell cytoskeleton through QCM-D technology is of pivotal importance in the monitoring of cell biological phenomena such as cell migration and cell differentiation. Data collected by the microgravimetric analyses using the QCM-D system could give information on biological mechanisms which are still difficult to predict. In this context platelets conformational modifications and cytoskeleton changes are involved in the platelet activation process, which is the major player in the formation of blood clotting on biomaterial surfaces. QCM-D was employed to evaluate how composition of bioengineered surfaces affected the platelet activation, which is crucial in the design of blood-contacting devices, as no activation is foreseen to avoid thrombogenic effects. Platelets activation was observed on fibronectin-coated sensors as fibronectin is a highly thrombogenic protein. As discussed above, Δ*f* alone does not give sufficient information and the analysis Δ*D-*Δ*f* plot is the more appropriate method to observe changes in cell shape. Δ*D-*Δ*f* plot revealed two phases in platelet activation process, a first phase corresponding to initial interactions among surface sensor and cells with a decrease of Δ*f* and a slight increase of Δ*D* followed by a second phase characterized by an increase of Δ*D* ascribed to the platelet spreading and pseudopodia formation (Figure 1B). The higher is the slope of the second phases, the lower is the thrombogenicity of the surface as a low anchorage of the platelets on the surfaces resulted in a less rigid layer (Fatisson et al., 2008). Assays on thrombogenicity of high-density polyethylene (HDPE) and polycarbonate (PC) were performed evaluating the effect of surface chemical composition, roughness, and wettability, on platelet activation highlighting that the hemocompatibility effect of these polymers was mainly due to their hydrophobic nature (Fatisson et al., 2011b).

QCM-D system was also applied by Zhang et al. (2015b) to study the difference between healthy erythrocytes (RBCs) and RBCs derived from type 2 diabetic patients, as vascular thrombosis is the most frequent complication in diabetic patients. Δ*D-*Δ*f* plot revealed that RBCs from diabetic individuals are stiffer and less prone to cytoskeleton rearrangements than healthy cells. Furthermore, RBCs were co-cultured for 30 min on a QCM-D sensor previously coated with HUVECs. QCM-D signals revealed that pathological RBCs adhered to HUVECs while no adhesion was observed for healthy RBCs.

Among other potential applications, QCM-D was exploited to give novel insights in the study of cancer cell motility which has been recognized as an essential value to quantify the metastatic potential of cancer cells (Friedl and Gilmour, 2009). Tarantola et al. (2010) proposed a QCM-based sensor to determine the motility of four breast cancer lines with different invasiveness by correlating frequency fluctuations recorded by QCM system to collective motility. High fluctuations were associated to collective mobility, which is typical of tumor progression phase. The higher was the fluctuation the more invasive was the cell population as confirmed by the traditionally used Boyden chamber method. This new approach allows measuring the invasiveness of tumor in colonizing adjacent connective tissues helping to identify the degree of tumor malignancy and to define the more appropriate treatment.

## Conclusion and future perspectives

The collected literature highlighted the role of QCM-D technology as a new and non-conventional facility to monitor cells in real time using a label-free methodology. Several phenomena can be detected from initial cell binding, focal adhesion formation, cell spreading, and complex changes in cytoskeleton. The attachment and growth of cells is the key step to achieve device biointegration and many efforts are addressed to the design of bioengineered surface able to enhance cell growth. *In vitro* and *in vivo* tests currently applied lack the ability to follow the initial phase of cell/surface interactions which can be monitored modeling QCM-D signals. Complex phenomena occurring during cell/external environment interactions can be distinguished and analyzed by combining temporal frequency and energy dissipation shifts in Δ*D-*Δ*f* plots.

To date, QCM-D is rarely applied in materials and surfaces characterization, however, the data collected from this measuring system are unique and could drive to a better understanding of biological phenomena which is fundamental to engineer devices with enhanced biological performances. In addition, taking advantage of the high sensitivity of QCM-D instruments in detecting tiny changes in cell conformation, it is possible to analyse the cytoskeleton changes induced by different drugs on pathological and healthy cells. Consequently, the more appropriate pharmacological treatment can be defined helping in developing methodologies for advanced and/or personalized therapies as well as screening tools. Furthermore, recent upgrades of the QCM-D device allow to match the recorded Δ*f* and Δ*D* signals with morphology and optical properties of studied platforms by connecting the sensor chamber with additional apparatus such as microscope and ellipsometer.

In conclusion, the recent literature clearly indicates that QCM-D is already a promising methodology to study cell behavior since it provides data which are pivotal to achieve a complete understanding of many biological phenomena consequently helping in tackling the current challenges in the biomedical field.

## Author contributions

CT-T revised the current literature on QCM-D and wrote the main text. IC summarized the state of the art focused on application of QCM-D in cytoskeleton changes analysis. GC revised the manuscript discussing the future trends of QCM-D applications.

### Conflict of interest statement

The authors declare that the research was conducted in the absence of any commercial or financial relationships that could be construed as a potential conflict of interest.
